# Dependence of Bone Yield (Volume of Bone Formed per Unit of Cement Surface Area) on Resorption Cavity Size During Osteonal Remodeling in Human Rib: Implications for Osteoblast Function and the Pathogenesis of Age-Related Bone Loss

**DOI:** 10.1359/jbmr.091003

**Published:** 2009-10-12

**Authors:** Shijing Qiu, D Sudhaker Rao, Saroj Palnitkar, A Michael Parfitt

**Affiliations:** 1 Bone and Mineral Research Laboratory, Henry Ford Hospital Detroit, MI, USA; 2 Division of Endocrinology and Center for Osteoporosis and Metabolic Bone Disease, University of Arkansas for Medical Sciences Little Rock, AR, USA

**Keywords:** haversian canal area, focal bone balance, bone remodeling, bone circulation, bone yield

## Abstract

It is both a necessary and a sufficient condition for bone to be lost with age at any surface location that during remodeling the replacement of resorbed bone is incomplete. In both the ilium and the rib, the degree of such focal imbalance is smaller on the intracortical than on the endocortical or cancellous surfaces that are adjacent to bone marrow. The reason for this difference is unknown. To further examine this question, we measured various geometric variables in 1263 osteons in rib cross sections from 65 persons, including both sexes and age ranges 20 to 30 years and 60 to 70 years (four groups). Haversian canal (HC) area did not differ significantly between sexes or age groups. Percent osteonal refilling was close to 95% in all groups and did not differ between sexes but fell slightly with age. There was a very highly significant linear relationship between osteon bone area and (osteon area + HC area) in all groups, with coefficients of determination (*r*^2^) greater than 0.98. The regression slopes declined slightly with age in women but not in men. There was a very highly significant quadratic relationship between osteon bone area and osteon perimeter in all groups, with *r*^2^ values greater than 0.97. The ratio osteon bone area:osteon perimeter, an index of bone yield—the volume of bone deposited on each unit area of cement surface—was strongly related to osteon area and did not differ between sexes but was slightly less in the older groups. We conclude the following: (1) The high efficiency of intracortical remodeling in the rib is confirmed, with only trivial effects of age. (2) For HC area to be maintained within narrow limits and bone balance preserved, either initial osteoblast density or osteoblast capacity (the two determinants of bone yield) or, most likely, both must increase progressively with the size of the resorption cavity, suggesting that osteoblast recruitment (relative to available surface) and osteoblast lifespan increase with the volume of bone resorbed. (3) Intracortical remodeling in the rib is more efficient than marrow-adjacent remodeling at any site, possibly because of the different relationships to the circulation. In osteonal remodeling, all molecules released from resorbed bone must travel past the sites of osteoblast recruitment and operation, but in hemiosteonal remodeling, some molecules may not be subject to this constraint. (4) If marrow-adjacent remodeling became as efficient as rib intracortical remodeling, age-related bone loss would cease to be an important medical problem. © 2010 American Society for Bone and Mineral Research

## Introduction

Loss of bone with increasing age has been an inescapable feature of human biology since prehistoric times.(1,2) This process cannot be fully understood without awareness of the structural changes that underlie but are not revealed by the usual densitometric measurements.(1,3) Except for the cranial vault, all bone lies between two envelopes, the outer one formed by the periosteum and articular cartilage and the inner one formed by the endosteum, which separates bone from bone marrow.(4) All age-related bone loss occurs from the endosteum, of which the three subdivisions—the cancellous, endocortical, and intracortical surfaces—are in continuity.(5) Each of these surfaces undergoes remodeling, the process mediated by basic multicellular units (BMU), whereby old bone is replaced by new bone.(4,6) During each focal remodeling transaction, the bone surface initially moves away from the adjacent soft tissue for a distance referred to as *resorption depth* (Rs.De) and then moves back toward the soft tissue for a distance referred to as *wall thickness* (W.Th). If, for whatever reason, W.Th. is less than Rs.De., replacement has been incomplete, and that transaction has resulted in irreversible bone loss, for which the focal imbalance is both a necessary and a sufficient condition.(1,6) This contrasts with reversible bone loss owing to expansion of the remodeling space.(7)

**Fig. 1:**
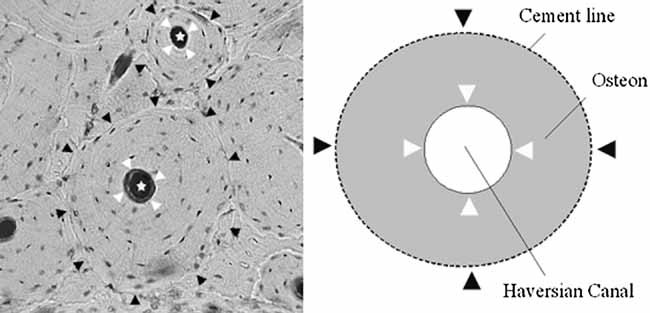
Left panel is a representative cross section of rib cortical bone. In two osteons of different size, the cement line, representing the furthest extent of resorption prior to construction of the osteon, is highlighted with black arrowheads, and the boundary of the haversian canal, representing the furthest extent of formation during construction of the osteon, is highlighted with white arrowheads. The right panel indicates schematically the basis of the primary measurements. The cement line is shown as an interrupted line and the haversian canal boundary as a solid line. Osteon area is the entire area circumscribed by the cement line, from which is subtracted the haversian canal area to obtain osteon bone area.

During early rapid bone loss from the cancellous surface, some trabeculae are completely removed because of increased Rs.De, leading to fenestration, and those remaining slowly become thinner because of reduced W.Th.(1,6) Loss of bone from the endocortical surface occurs by enlargement and confluence of subendocortical cavities that are extensions of the marrow cavity; this process, known as *cancellization*, is the result of increased Rs.De that persists throughout life so that bone cortices become progressively thinner.(8) Rs.De depends mainly on the lifespan of osteoclasts, which is determined by the timing of osteoclast apoptosis.(9) Changes at the intracortical surface are more subtle. Cortical bone, often called *compact*, is mainly solid but is traversed by small channels called *haversian canals* at the center of each osteon that contain blood vessels and nerves. The canals are about 50 µm in diameter and in aggregate cross-sectional area (cortical porosity) occupy about 5% of the total area (Fig. 1). With age, there is a small increase in cortical porosity in the ilium (from 4.8% to 6.2% in healthy women) owing mainly to an increase in the number of haversian canals.(10,11) When a new canal is made, W.Th is inevitably less than Rs.De, lest there be no room for the canal contents. There is also a small increase in canal diameter with age owing mainly to increased Rs.De in men and decreased W.Th in women.(12) The relative contributions of the three endosteal subdivisions to bone loss in the ilium are approximately 53%, 40%, and 7%, respectively.(10)

Loss of cancellous bone owing to removal of trabeculae and loss of cortical bone owing to increased endocortical resorption also occur in the spine and in the femur and most likely in every other bone,1 but the changes at the intracortical surface vary more between bones. In the femur, the increase in cortical porosity is greater than in the ilium, reaching values above 15% to 20% in many people after age 60(13); there is no increase in osteonal size (area in section) but incomplete refilling owing to a fall in W.Th.(14) By contrast, in the rib, there is a fall in osteonal size but no significant change in haversian canal size,(15) so focal bone balance is better preserved in the rib than in either the femur or the ilium.

Bone histomorphometry is performed on two-dimensional (2D) microscopic sections, but it is important always to consider how these measurements relate to three-dimensional (3D) reality.(16) Newly formed bone structural units are demarcated from older bone by the cement line, which corresponds in 3D to the surface on which cement substance was deposited after the resorption cavity has been fully excavated. All new bone formation in the uninjured adult skeleton begins on the cement surface, where all newly formed osteoblasts are assembled.(4,6) The amount of bone formed on each unit area of the cement surface, of which mean W.Th is an estimate, is of critical importance for the maintenance of bone balance but currently lacks a convenient name. By analogy with crop production in bushels per acre, we will refer to it as *yield*. Both farmland yield and bone yield are expressed in units of volume per area.

Bone yield depends on the number of osteoblasts recruited per unit cement surface area, referred to as *initial osteoblast density*, and on the total amount of bone made by each osteoblast during its lifespan, referred to as *osteoblast capacity*.(17) *Osteoblast density* is the inverse of osteoblast secretory territory—the area over which each osteoblast needs to deposit new bone matrix.(18) The smaller this area, the greater is the contribution to cavity repair and wall thickness made by the same amount of bone matrix production by each osteoblast. When osteoblasts are tall and columnar in shape, their secretory territory is low, and as they become thinner and flatter, their secretory territory increases.(17,18) Osteoblast capacity is the product of matrix production rate, of which mean mineral apposition rate is the best estimate,(17) and osteoblast lifespan. Wall thickness depends not only on these variables but also on a dimensionness shape factor that corrects for differences in curvature between different surfaces.(17)

In a preliminary study of the human rib confined to young men, we found that although osteons vary greatly in cross-sectional area, the extent of fractional refilling is much less variable.(19) Here we report more extensive measurements in a larger sample that includes both sexes and older as well as younger persons that demonstrate that bone yield, which is determined by initial osteoblast density and osteoblast capacity, increases in proportion to the size of the resorption cavity. The rib was the first site used for dynamic bone histomorphometry in living human subjects,(20) but it is by no means representative of the whole skeleton—for example, it is the only bone that is never at rest. Nevertheless, our data that address the mechanism for the preservation of focal bone balance in the rib cortex indicate what the remodeling apparatus can accomplish in ideal circumstances and may lead to a better understanding of why focal bone balance becomes negative with age at other sites and in other bones.

## Materials and Methods

### Specimen preparation

Archived human rib sections were used for this study. The ribs were obtained from Caucasian subjects either at autopsy of otherwise healthy persons who died suddenly or at thoracotomy for diseases not known to affect bone. There were 65 subjects, 23 men and 14 women, aged 20 to 30 years and 14 men and 14 women aged 60 to 70 years. The method of sample processing has been described previously.(21) In brief, the 3 inch rib segments were placed in 1% basic fuchsin and 40% ethyl alcohol for 4 weeks and then immersed in a large volume of tap water for 48 hours. After hydration, cross sections were cut from each rib, ground to a thickness of about 50 µm, and mounted on a slide.

### Histomorphometry

Microscopy of osteons was performed on the cross sections using a Nikon microscope equipped with a CCD video camera (Optronics, Goleta, CA, USA). The microscopic image was imported to a Bioquant NOVA image analysis system (R&M Biometrics, Inc., Nashville, TN, USA) with a panel size of 640 × 480 pixels. All the secondary osteons that met the following criteria were examined: (1) an intact osteon with a clear cement line boundary, (2) no evidence of remodeling, either resorption or formation (because thin osteoid seams may escape detection, we excluded osteons in which the haversian canal area was larger than one-quarter of the osteon area; although this criterion may exclude some osteons in which bone formation ceased prematurely, such osteons, although quite common in the femur,(13,14) are extremely rare in the rib(15)), and (3) no Volkmann's canals crossing the osteon. Based on these criteria, we measured 1263 osteons from young men, 724 from young women, 1087 from older men, and 944 from older women; the number in each subject ranged from 34 to 148, and the mean numbers per subject were about 55, 52, 78, and 67, respectively, in the four groups. As depicted in Fig. 1, osteon area (On.Ar) and perimeter (On.Pm) were measured by tracing the cement line, and haversian canal area (HC.Ar) and perimeter (HC.Pm) were measured by tracing the boundary of the haversian canal. All measurements were performed under brightfield microscopy using a 10× objective. From these data we calculated osteon bone area (On.B.Ar = On.Ar – HC.Ar), the percentage of osteon refilling (On.B.Ar/On.Ar × 100), and the ratio between On.B.Ar and On.Pm (On.B.Ar/On.Pm).

### Statistics

The focus of our study was osteonal remodeling and the effects on this process of age and sex, so the unit of observation for all regression analyses was the osteon. However, we calculated descriptive statistics for each variable with the study subject as the unit of observation.

Two-way ANOVA was used to compare the difference in each variable between groups of different sexes and ages. Analysis of covariance (ANCOVA) was performed to compare the difference in On.B.Ar/On.Pm by adjusting for its regression on On.Ar. Best-fitting nonlinear as well as linear regressions were used for testing the relationship in each group between the calculated variables and both On.Pm and On.Ar as independent variables. The relationship between cavity size (On.Ar) and the amount of bone replaced (On.Ar – HC.Ar) is complicated by the common variable On.Ar. As shown by Oldham,(22) the difference between two values (*x*_1_ – *x*_2_) is always positively correlated with *x*_1_ and negatively correlated with *x*_2_ even if taken from a table of random numbers. When *x*_1_ and *x*_2_ represent successive measurements in the same subject, this problem can be circumvented by using the mean of *x*_1_ and *x*_2_ rather than *x*_1_ as the independent variable.(22) We have applied this method to avoid artifactual correlation, but to maintain the appropriate scale, we used the sum of On.Ar and HC.Ar rather than their mean.(22)

## Results

The primary measured and calculated data in the four groups are compared in Table 1. Osteon area and perimeter are both larger in men than in women in both age groups and are smaller in old than in young subjects in both sexes. Osteon bone area showed very similar effects of both sex and age. In contrast, haversian canal area showed no significant effect of either sex or age, although it was smaller in younger women than in younger men and larger in older women than in older men, a significant interaction. With pairwise testing, the value was slightly but significantly higher in young men than in old men or in young women. The extent of refilling was close to 95% in all groups but was slightly but significantly lower in the older than in the younger groups. The difference between sexes was not significant, but the reduction with age was 0.39% in men and 1.59% in woman, a significant interaction. The coefficient of variation (CV = SD/mean × 100) was approximately 10% for the perimeter measurement and approximately 20% for the area measurements but only about 1% for refilling.

**Table 1:** Variables for Osteon Morphology in the Four Groups

Variable	Young Men	Young Women	Old Men	Old Women	*p*^a^	*p*^b^	*p*^c^
*n*	23	14	14	14			
Mean Age (y)	25.1 (3.71)	26.1 (4.34)	65.1 (3.55)	65.2 (4.06)			
On.Pm (mm)	0.706 (0.070)	0.660 (0.035)	0.637 (0.056)	0.596 (0.059)	<0.001	0.005	0.870
CV (%)	9.9	5.4	8.8	9.8			
On.Ar (mm^2^)	0.0371 (0.0070)	0.0312 (0.0037)	0.0290 (0.0049)	0.0262 (0.0052)	<0.001	0.003	0.278
CV (%)	18.9	11.9	16.9	19.8			
HC.Ar (mm^2^)	0.0017 (0.0003)	0.0013 (0.0003)	0.0014 (0.0003)	0.0016 (0.0005)	0.924	0.268	0.005
CV (%)	17.1	23.1	21.4	31.2			
OnB.Ar (mm^2^)	0.0354 (0.0067)	0.0299 (0.0036)	0.0276 (0.0048)	0.0247 (0.0048)	<0.001	0.003	0.347
CV (%)	18.9	12.0	17.4	18.6			
OnB.Ar/On.Ar (%)	95.3 (0.43)	95.5 (0.86)	94.9 (1.15)	94.0 (1.03)	<0.001	0.084	0.008
	0.45	0.91	1.21	1.09			

Data expressed as mean (SD) CV = coefficient of variation (SD/mean *100)

a *p* value for age

b *p* value for sex

c *p* value for interaction.

**Fig. 2:**
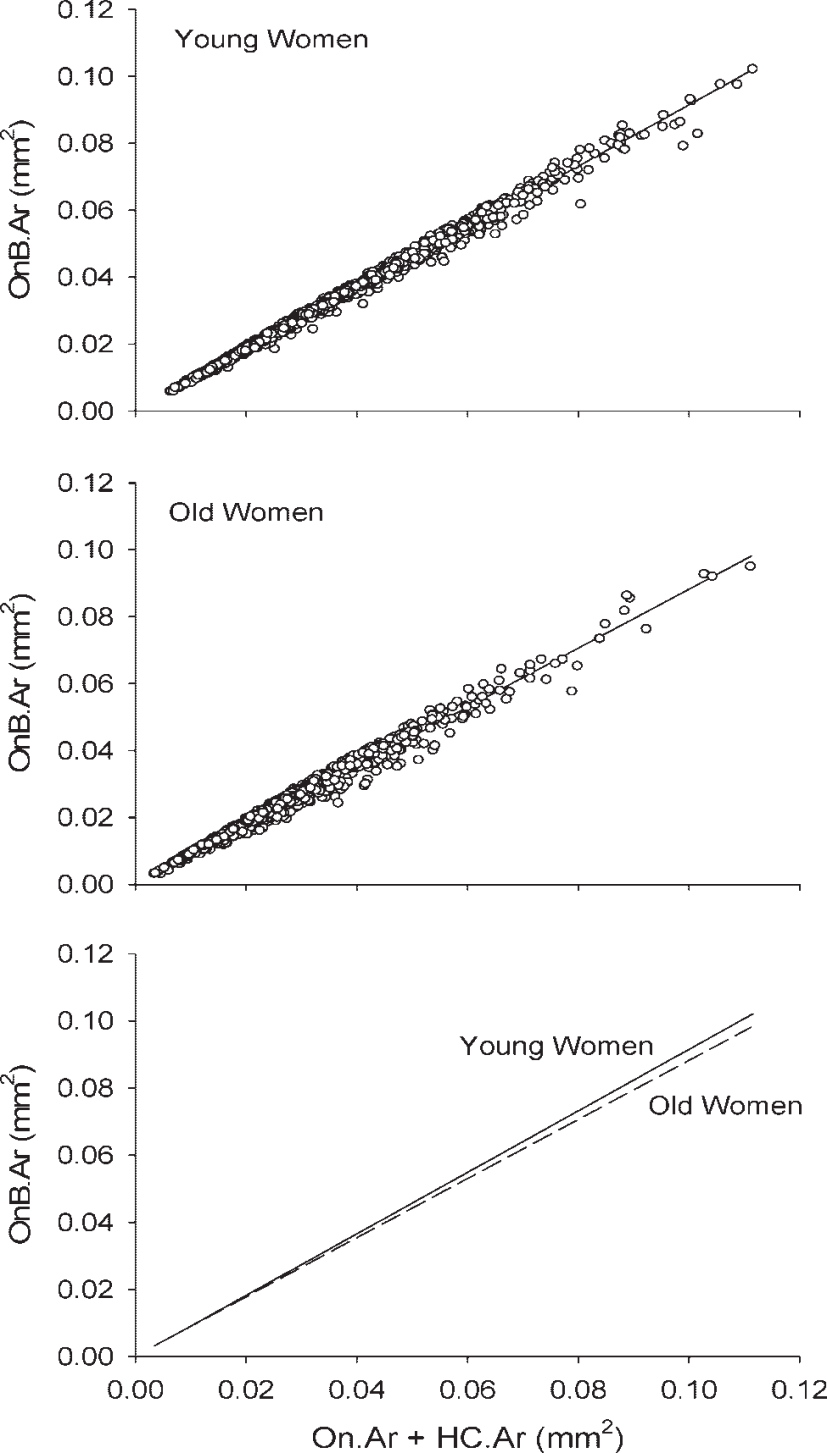
The relationship between On.B.Ar and (On.Ar + HC.Ar) in young (*upper panel*) and old (*middle panel*) women, with regression slopes compared in the lower panel. The regression slopes for old and young men lay between these (see Table 2). Each data point represents a single osteon.

In each demographic group there was a very highly significant (*p* < .0001) linear regression of On.B.Ar on (On.Ar + HC.Ar) (Fig. 2 and Table 2). The slopes ranged from 0.88 to 0.93, in agreement with the small variation in percent refilling (see Table 1). The regression intercepts were not significantly different from 0, and the values for *r*^2^ were 0.98 or higher, so (On.A + HC.Ar) accounted for more than 98% of the variance in On.B.Ar, and all other factors together accounted for less than 2%. The two most different slopes are shown in Fig. 1, and the four slopes are compared in Table 2. In men, the slopes did not vary significantly with age, but in women, the slope was slightly but significantly lower in the older than in the younger group, in keeping with the data in Table 1. The slope was significantly higher in young women than in young men but significantly lower in old women than in old men. There also were significant relationships for both HC.Ar and On.B.Ar/On.Ar with On.Ar, but the values for *r*^2^ were much lower (data not shown).

**Table 2:** Regression of OnB.Ar on (On.Ar + Hc.Ar) in the Four Groups

			Age		
				
			Young	Old	p^a^
		Slope	0.934	0.880	<0.001
	Female				
		*r*^2^	0.989^b^	0.983	
Sex					
		Slope	0.915	0.907	NS
	Male				
		*r*^2^	0.994^b^	0.985	
	*p*^c^		<0.001	<0.001	

a For effects of age on slope.

b Coefficient of determination = explained variance/total variance.

c For effects of sex on slope.

**Fig. 3:**
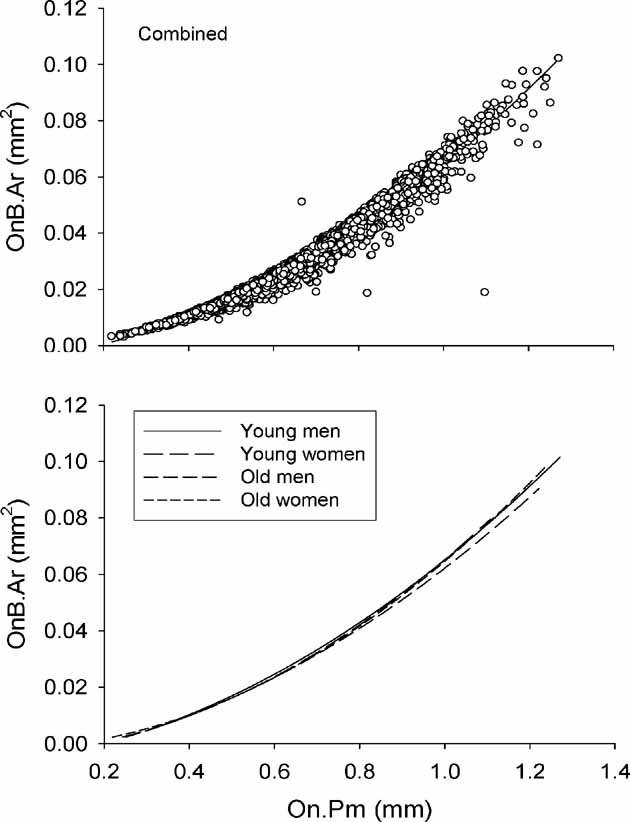
The quadratic relationship between On.B.Ar and On.Pm. In the upper panel are shown the data for all subjects; two outlying points were derived from elliptical cross sections that change the relationship between On.Ar and On.Pm. The quadratic regression equation is On.B.Ar = –0.0056 + 0.01181 × On.Pm + 0.0526 × On.Pm^2^; *r*^2^ = 0.972, *p* < .0001. In the lower panel the quadratic regression lines of the four groups are compared. Each data point represents a single osteon.

**Fig. 4:**
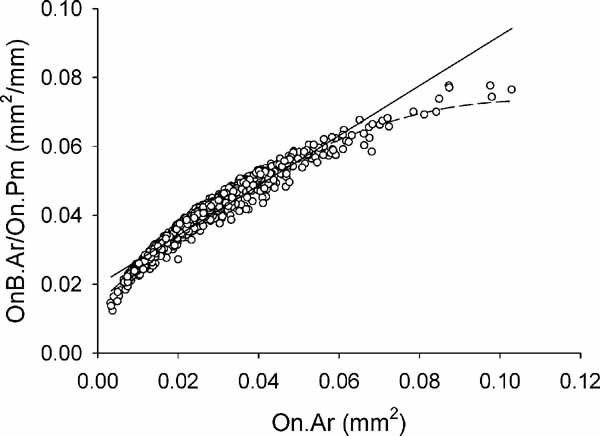
Linear (*solid line*) and curvilinear (*interrupted line*) relationships between On.B.Ar/On.Pm and On.Ar in old women. The regression equations, both linear and curvilinear were very similar in the other demographic groups. Each data point represents a single osteon.

In each demographic group there was a very highly significant (*p* < .0001) quadratic regression of On.B.Ar on On.Pm (Fig. 3); the regression parameters were very similar among the four groups. The regression intercepts were not significantly different from 0, and the values for *r*^2^ were 0.97 or higher. so On.Pm accounted for more than 97% of the variance in On.B.Ar, and all other factors together accounted for less than 3%. Because of this close relationship, it is of interest to compare values for the ratio On.B.Ar/On.Pm between groups (Table 3). The mean value was significantly lower in older than in younger subjects in both sexes and significantly lower in women than in men in both age groups. However, there was a significant (*p* < .0001) relationship, both linear and curvilinear, between On.B.Ar/On.Pm and On.Ar in all demographic groups (Fig. 4). When the values for the ratio were adjusted for the linear relationship in each group by analysis of covariance, the effect of sex was eliminated, and the effect of age was markedly attenuated, falling with age by about 2% instead of by about 12%, but remained significant (Table 3).

**Table 3:** Unadjusted and Adjusted Values for OnB.Ar/On.Pm

Variable	Young Men	Young Women	Old Men	Old Women	*p*^a^	*p*^b^	*p*^c^
OnB.Ar/On.Pm (mm^2^/mm)	0.0472 (0.0049)	0.0432 (0.0031)	0.0408 (0.0038)	0.0386 (0.0041)	<0.001	0.005	0.394
OnB.Ar/On.Pm (mm^2^/mm)^d^	0.0433 (0.0008)	0.0436 (0.0007)	0.0428 (0.0007)	0.0427 (0.0008)	<0.001	0.651	0.190

Data expressed as mean (SD).

a *p* value for age

b *p* value for gender

c *p* value for interaction

d Values adjusted for On.Ar in each group by ANCOVA.

## Discussion

In the adult human skeleton, bone remodeling serves to replace damaged or effete bone as exactly as possible, and to accomplish this purpose, new bone must be formed at the right time and place and in the right amount.(1,6) The usually small difference between *mean* values of Rs.De and W.Th(6) could conceal a wide variation in the magnitude and direction of focal bone balance at different locations. In cancellous bone, a close focal matching of W.Th to Rs.De can be inferred from the preservation of a generally smooth surface without abrupt elevations or depressions but is impossible to verify with certainty because the original location of the surface before the remodeling cycle was begun can only be inferred indirectly.(23,24) However, the different geometry of intracortical remodeling has enabled us to demonstrate unambiguously an extremely close relationship between the depth of an individual resorption site and the amount of bone laid down at that location. Since such close matching is essential to its function, it is likely to be a universal feature of the remodeling system, except where the template for bone formation has been destroyed by focally excessive resorption.(6)

We found only a modest effect of increased age. In all subjects, the mean value of On.B.Ar/On.Ar declined by only about 1% (see Table 1) and the mean value of On.B.Ar/On.Pm declined by only about 2% (see Table 2) over a mean age difference of 39.7 years. Although statistically significant, these changes were insufficient to produce a significant increase in HC.Ar. This was partly the result of a significant age-related reduction in On.Ar, so the task for osteoblasts was made easier by reducing the size of resorption cavities. Evidently, the maintenance of bone balance is more efficient for intracortical remodeling in the rib than for endocortical or cancellous remodeling at any site. On these surfaces, although individual values for Rs.De and W.Th would be highly correlated, the difference between them, an expression of focal bone balance, would be greater than on the intracortical surface. HC.Ar, whether expressed as an absolute value or as a fraction, differed only slightly between subjects (see Table 1), and if taken as constant, all the relationships we have found could be inferred. Nevertheless, it is pertinent to consider how such constancy could be achieved—what has to happen at the cellular level and how the necessary changes in cell function are produced.

**Fig. 5:**
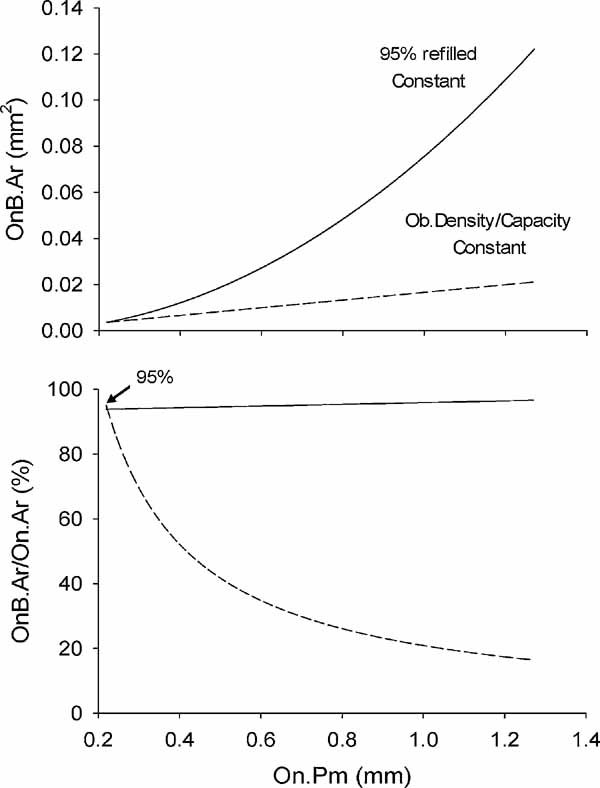
Consequences of constant amount of bone made per unit cement surface area (constant bone yield because of constant osteoblast secretory territory and lifespan). In the upper panel, if osteons are 95% refilled (see Table 1), the relationship between OnB.Ar and On.Pm (*upper solid line*) is quadratic—proportional to On.Pm^2^, as in Fig. 3. If bone yield is constant, the relationship (*lower interrupted line*) is linear—proportional to On.Pm. The lower panel shows the observed relationship between percent refilling and On.Pm (*upper solid line*) compared with the relationship corresponding to constant bone yield (*lower interrupted line*).

Further interpretation of our results requires an understanding of the relationship between measurements made on a 2D section and the 3D reality. In the long bones of the extremities, the long axis of the osteons rarely departs by more than 10 degrees from the neutral axis,(25) and for the most part, the same applies to the rib.(4,15) Consequently, the area:perimeter ratios we report can be converted directly to volume:surface ratios without the need to correct for section obliquity.(16) Accordingly, as explained in Fig. 5, we have demonstrated that bone yield (volume of bone made on each unit area of cement surface available for osteoblast recruitment) increases directly with the size of the resorption cavity and hence with the volume of bone resorbed. Furthermore, these relationships differ only trivially between men and women and between young and old persons. If bone yield remained constant, then with increasing resorption cavity size, percent refilling would decline and HC.Ar would increase (see Fig. 5). In order to maintain HC.Ar within narrow limits and so preserve bone balance, either initial osteoblast density or osteoblast capacity as previously defined (the two determinants of bone yield)(17) or both must increase with the size of the resorption cavity.

Despite its importance for the preservation of bone balance, initial osteoblast density rarely has been measured. Marotti(26) reported a mean value of 6500/mm^2^ (corresponding to a secretory territory of 153 µm^2^/osteoblast) in dog cortical bone but gave few details. A similar value was found in growing rat parietal bone,(18) which represents modeling rather than remodeling. In human rib, the mean value was 4500/mm^2^ of osteoid seam surface area, a secretory territory of 222 µm^2^/osteoblast,(27) but the distance from the cement surface could not be determined. There was an approximately threefold difference between the lowest and highest values (see Table 3), sufficient to account for part of the variability in On.B.Ar/On.Pm. However, from the curvilinear regression (see Fig. 4), there was an approximately sixfold difference between the lowest and highest values, too large to be accounted for only by variation in initial osteoblast density. Osteoblast capacity as defined has never been measured but is most likely determined mainly by osteoblast lifespan and hence by the timing of osteoblast apoptosis.(6,17) Both initial osteoblast recruitment(28) and the timing of osteoblast apoptosis(29) could be influenced by factors released from resorbed bone, of which the concentration would be influenced by the volume of bone resorbed, a concept incorporated into a recently developed mathematical model of bone remodeling.(30)

**Fig. 6:**
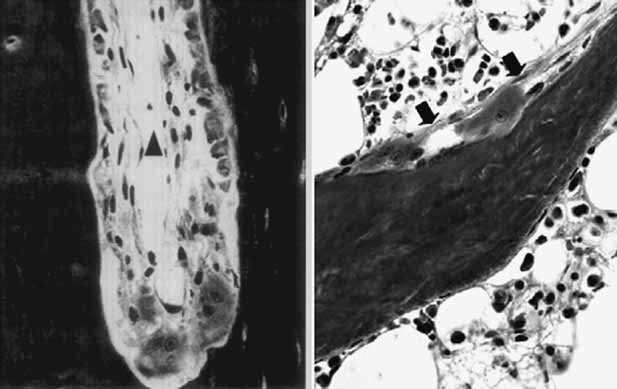
On the left, cortical BMU indicating short distance between osteoclasts and site of osteoblast recruitment and presence of capillary (*arrowhead*) able to carry molecules to older osteoblasts that are more distant. (Reprinted from ref. (32) with permission.) On the right, cancellous bone remodeling compartment showing osteoclasts separated from the marrow only by a canopy of former lining cells (*arrows*). (Reprinted from ref. (34) with permission, courtesy of Robert S Weinstein, MD.)

One possible explanation for the greater efficiency of intracortical (osteonal) remodeling than of remodeling of bone adjacent to the bone marrow (hemiosteonal) is the different relationships to the circulation.(31–34) Because of the 3D organization of an intracortical BMU (Fig. 6*A*), all molecules released by bone resorption in the cutting cone must travel past the sites of bone formation in the closing cone. Osteoblast recruitment occurs sufficiently close to the cutting cone that molecules released from resorbed bone could travel to the site of recruitment by simple diffusion. Older osteoblasts facing a choice between continued matrix synthesis, transformation to osteocytes, or death by apoptosis are much further from the cutting cone but could be influenced by molecules carried in the efferent limb of the capillary. Much less is known about the circulatory component of remodeling adjacent to marrow,(33) but the so-called bone remodeling compartment (see Fig. 6*B*) appears to allow for some molecules released from resorbed bone to reach the marrow circulation directly, without having the opportunity to influence bone formation. Furthermore, reciprocal signaling between osteoclasts and osteoblasts, such as that mediated by the ephrin system,(35) would be more effective with a closed rather than an open circulation.

Another possible explanation for the difference in remodeling efficiency is a difference in geometry. During intracortical remodeling, osteonal refilling leads to a *decline* in the radius of curvature, but in marrow-adjacent remodeling, refilling generally leads to an *increase* in the radius of curvature. Conceivably, curvature-related differences in surface strain might influence osteoblast recruitment, and changes in surface strain or cell membrane stress might influence osteoblast lifespan. Since strain mediates the beneficial effect of physical activity on bone,(36) the constant movement of the ribs during respiration might play a role. The small variation in percent refilling (see Table 1) raises the possibility that toward the end of their lifespan osteoblasts may be able to sense the size of the canal or the closeness of osteoblasts on the other side of the canal. Although HC.Ar was significantly related to On.Ar, the unexplained variance ranged from 47% to 75%, and the CV of HC.Ar ranged from 17% to 33% (see Table 1). Nevertheless, there might be other differences between the closed circulation of intracortical remodeling and the open circulation of the bone marrow, such as pressure, fluid flow, or oxygen tension, that are important. Finding the reason would be useful because it eventually could explain why focal balance worsens progressively with increasing age in the femur, where the most clinically important fractures occur, whereas it is maintained so well in the rib. Furthermore, if marrow-adjacent remodeling became as efficient as intracortical remodeling in the rib, osteoporosis would no longer be a significant medical problem.

## References

[1] Parfitt AM, Compston J, Ralston SH (2000). Osteoporosis: 50 years of change, mostly in the right direction. Osteoporosis and Bone Biology: The State of the Art.

[2] Perzigian AJ (1973). Osteoporotic bone loss in two prehistoric Indian populations. Am J Phys Anthropol..

[3] Parfitt AM http://www.uninet.edu/cin2003/conf/parfitt.html.

[4] Frost HM (1966). The Bone Dynamics in Osteoporosis and Osteomalacia.

[5] Parfitt AM, Kleerekoper M, Krane SM (1989). Surface specific bone remodeling in health and disease. Clinical Disorders of Bone and Mineral Metabolism.

[6] Parfitt AM, Agarwal SC, Stout SD (2003). New concepts of bone remodeling: a unified spatial and temporal model with physiological and pathophysiologic implications. Bone Loss and Osteoporosis: An Anthropological Perspective.

[7] Heaney RP (1994). The bone-remodeling transient: implications for the interpretation of clinical studies of bone mass change. J Bone Miner Res..

[8] Han ZH, Palnitkar S, Rao DS, Nelson D, Parfitt AM (1997). Effects of ethnicity and age or menopause on the remodeling and turnover of iliac bone: implications for mechanisms of bone loss. J Bone Miner Res..

[9] Parfitt AM, Mundy GR, Roodman GD, Hughes DE, Boyce BF (1996). A new model for the regulation of bone resorption, with particular reference to the effects of bisphosphonates. J Bone Miner Res..

[10] Han ZH, Palnitkar S, Rao DS, Nelson D, Parfitt AM (1996). Effect of ethnicity and age or menopause on the structure and geometry of iliac bone. J Bone Miner Res..

[11] Wu K, Schubeck KE, Frost HM, Villanueva A (1970). Haversian bone formation rates determined by a new method in a mastodon, and in human diabetes mellitus and osteoporosis. Calcif Tissue Res..

[12] Brockstedt H, Kassem M, Eriksen EF, Mosekilde L, Melsen F (1993). Age- and sex-related changes in iliac cortical bone mass and remodeling. Bone..

[13] Jowsey J (1960). Age changes in human bone. Clin Orthop..

[14] Jowsey J (1966). Studies of haversian systems in man and some animals. J Anat..

[15] Landeros O, Frost HM (1964). The cross section size of the osteon. Henry Ford Hosp Med Bull..

[16] Parfitt AM, Recker R (1983). The stereologic basis of bone histomorphometry: theory of quantitative microscopy and reconstruction of the 3rd dimension. Bone Histomorphometry: Techniques and Interpretations.

[17] Parfitt AM, Hall BK (1990). Bone-forming cells in clinical conditions. Bone, Vol. 1: Osteoblast and Osteocyte.

[18] Jones SJ (1974). Secretory territories and rate of matrix production of osteoblasts. Calcif Tissue Res..

[19] Qiu S, Fyhrie DP, Palnitkar S, Rao DS (2003). Histomorphometric assessment of haversian canal and osteocyte lacunae in different-sized osteons in human rib. Anat Rec..

[20] Frost HM (1968). Tetracycline bone labeling in anatomy. Am J Phys Anthropol..

[21] Frost HM (1960). Presence of microscopic cracks in vivo in bone. Bull Henry Ford Hosp..

[22] Oldham PD (1962). A note on the analysis of repeated measurements of the same subjects. J Chronic Dis..

[23] Cohen-Solal ME, Shih MS, Lundy MW, Parfitt AM (1991). A new method for measuring cancellous bone erosion depth: application to the cellular mechanisms of bone loss in postmenopausal osteoporosis. J Bone Miner Res..

[24] Croucher PI, Garrahan NJ, Mellish RW, Compston JE (1991). Age-related changes in resorption cavity characteristics in human trabecular bone. Osteoporos Int..

[25] Hert J, Fiala P, Petrtyl M (1994). Osteon orientation of the diaphysis of the long bones in man. Bone..

[26] Marotti G, Meunier PJ (1977). Decrement in volume of osteoblast during osteon formation and its effect on the size of the corresponding osteocytes. Bone Histomorphometry: Second International Workshop.

[27] Schen S, Villanueva AR, Frost HM (1965). Number of osteoblasts per unit area of osteoid seam in cortical human bone. Can J Physiol Pharmacol..

[28] Dempster DW, Zhou H, Seibel MJ, Robins SP, Bilezikian JP (2006). New concepts in bone remodeling. Dynamics of Bone and Cartelage Metabolism: Principles and Clinical Applications.

[29] Hulley P, Russell G, Croucher P, Seibel MJ, Robins SP, Bilezikian JP (2006). Growth factors. Dynamics of Bone and Cartilage Metabolism.

[30] Pivonka P, Zimak J, Smith DW (2006). Model structure and control of bone remodeling: a theoretical study. Bone..

[31] Parfitt AM (1994). Osteonal and hemi-osteonal remodeling: the spatial and temporal framework for signal traffic in adult human bone. J Cell Biochem..

[32] Parfitt AM (2000). The mechanism of coupling: a role for the vasculature. Bone..

[33] Parfitt AM (2001). The bone remodeling compartment: a circulatory function for bone lining cells. J Bone Miner Res..

[34] Parfitt AM (2006). Misconceptions V: activation of osteoclasts is the first step in the bone remodeling cycle. Bone..

[35] Zhao C, Irie N, Takada Y (2006). Bidirectional ephrinB2-EphB4 signaling controls bone homeostasis. Cell Metab..

[36] Bonewald LF (2007). Osteocytes as dynamic multifunctional cells. Ann NY Acad Sci..

